# The Effect of the Stretch-Shortening Cycle in the Force–Velocity Relationship and Its Association With Physical Function in Older Adults With COPD

**DOI:** 10.3389/fphys.2019.00316

**Published:** 2019-03-26

**Authors:** Roberto Navarro-Cruz, Julian Alcazar, Carlos Rodriguez-Lopez, Jose Losa-Reyna, Ana Alfaro-Acha, Ignacio Ara, Francisco J. García-García, Luis M. Alegre

**Affiliations:** ^1^GENUD Toledo Research Group, Universidad de Castilla-La Mancha, Toledo, Spain; ^2^CIBER of Frailty and Healthy Aging (CIBERFES), Madrid, Spain; ^3^Department of Geriatrics, Hospital Virgen del Valle, Complejo Hospitalario de Toledo, Toledo, Spain

**Keywords:** aging, muscle power, resistance training, concentric, eccentric, potentiation

## Abstract

This study aimed to evaluate the effect of the stretch-shortening cycle (SSC) on different portions of the force–velocity (F–V) relationship in older adults with and without chronic obstructive pulmonary disease (COPD), and to assess its association with physical function. The participants were 26 older adults with COPD (79 ± 7 years old; FEV_1_ = 53 ± 36% of predicted) and 10 physically active non-COPD (77 ± 4 years old) older adults. The F–V relationship was evaluated in the leg press exercise during a purely concentric muscle action and compared with that following an eccentric muscle action at 10% intervals of maximal unloaded shortening velocity (V_0_). Vastus lateralis (VL) muscle thickness, pennation angle (PA), and fascicle length (FL) were assessed by ultrasound. Habitual gait speed was measured over a 4-m distance. COPD subjects exhibited lower physical function and concentric maximal muscle power (P_max_) values compared with the non-COPD group (both *p <* 0.05). The SSC increased force and power values among COPD participants at 0–100 and 1–100% of V_0_, respectively, while the same was observed among non-COPD participants only at 40–90 and 30–90% of V_0_, respectively (all *p* < 0.05). The SSC induced greater improvements in force, but not power, among COPD compared with non-COPD subjects between 50 and 70% of V_0_ (all *p* < 0.05). Thus, between-group differences in muscle power were not statistically significant after the inclusion of the SSC (*p* > 0.05). The SSC-induced potentiation at 50–100% of V_0_ was negatively associated with physical function (*r* = -0.40–0.50), while that observed at 80–100% of V_0_ was negatively associated with VL muscle thickness and PA (*r* = -0.43–0.52) (all *p* < 0.05). In conclusion, older adults with COPD showed a higher SSC-induced potentiation compared with non-COPD subjects, which eliminated between-group differences in muscle power when performing SSC muscle actions. The SSC-induced potentiation was associated with lower physical function, VL muscle thickness, and VL PA values. The SSC-induced potentiation may help as a compensatory mechanism in those older subjects with a decreased ability to produce force/power during purely concentric muscle actions.

## Introduction

In the last few decades, both life expectancy and the number of years of living with disability have increased among older adults (Crimmins et al., 2016). This increasing proportion of people over 65 years old could lead to a higher incidence of chronic degenerative diseases and to a greater demand for health and social care with a consequent impact on health spending (Lopreite and Mauro, 2017).

Muscle power is recognized as a major target for resistance training interventions conducted to improve physical function in older adults (Sayers, 2008; Reid and Fielding, 2012; Izquierdo and Cadore, 2014). The capacity to produce muscle power is mediated by the ability to exert force and velocity, and thus it depends on the individual force–velocity (F–V) relationship. The evaluation of the F–V profile in older people has been shown to be a novel and effective strategy to differentiate between subjects with different functional states (Alcazar et al., 2018), and to individualize exercise programs in order to improve physical performance and enhance muscle power production in young people ( Samozino et al., 2014; Jimenez-Reyes et al., 2016). The F–V profile is commonly evaluated by recording the velocity exerted with increasing isotonic loads or force produced at varying isokinetic velocities (Alcazar et al., 2017). Both methods provide information about the neuromuscular status of the subjects being measured; however, isotonic recordings may be preferred in older people because of their similarity to the movements they perform in daily life. The evaluation of the F–V relationship has been shown to be reliable in older people (Alcazar et al., 2017). This method to assess neuromuscular function in older populations might provide us with several advantages over other methods [such as the one repetition maximum (1RM) test] in terms of relevant information on the force and velocity components of muscle power production (Alcazar et al., 2018).

The stretch-shortening cycle (SSC) is a phenomenon characterized by the enhancement of power production in a concentric muscle action preceded by active muscle stretching (eccentric muscle action) in comparison with the same concentric action preceded by a resting state. The SSC can improve muscle power production by 50% in some tasks such as vertical jumping (Bosco et al., 1982b). The known enhancement of performance due to the inclusion of the SSC has been attributed to the storage of elastic energy in the muscle during the stretch and its re-use as mechanical work during the concentric phase when it follows immediately (Bosco et al., 1982b). Reflexively induced neural input has also been suggested to be involved in the enhanced muscle function derived from a SSC action (Komi and Bosco, 1978; Bosco et al., 1982a; Komi, 1984).

However, while the SSC has been proven to increase neuromuscular performance in young adults (Komi, 1984; Jiménez-Reyes et al., 2014; Pallarés et al., 2014), some discrepancies exist regarding older people. In the aged population, a SSC-related increment in mechanical performance has been reported in some studies (Svantesson and Grimby, 1995; Lindle et al., 1997), although it has also been found to be diminished in comparison with younger subjects (Bosco and Komi, 1980). Interestingly, Izquierdo et al. (1999) found a decreased performance on the bench press exercise after a SSC compared to a solely concentric action in middle-aged and older men, although a greater jump height was achieved after the inclusion of the SSC. Another study observed a significant increase in jump height induced by the SSC in young subjects, but not in the middle-aged and older groups (Paasuke et al., 2003). By contrast, a SSC-induced potentiation was observed during plantar flexions in middle-aged and elderly subjects, without any age-related difference in comparison with young subjects (Svantesson and Grimby, 1995). The discrepancies across studies might be caused by the different muscles being evaluated (e.g., upper vs. lower limbs) and/or the differences among the aged populations being included (e.g., active vs. sedentary older adults). However, there are no studies evaluating the functional relevance that SSC-induced potentiation has in older people. Furthermore, limb muscle dysfunction is a prevalent condition in older people with chronic obstructive pulmonary disease (COPD), and is characterized by the presence of a decreased proportion of type I muscle fibers and oxidative capacity, and reduced muscle cross-sectional area, strength, and endurance (Maltais et al., 2014). The evaluation of the influence of the SSC on muscle performance in these patients might contribute to the understanding of this COPD-related myopathy.

Thus, our main goals were to assess the ability of the SSC to enhance muscle power during concentric actions in older people with COPD, and to evaluate the association between SSC-induced power potentiation and physical function and the influence of muscle size and architecture in older people with COPD.

## Materials and Methods

### Subjects

Twenty-six outpatients with COPD (FEV1 = 53.4 ± 35.6% of predicted; BODE index = 3.9 ± 2.1) and 10 physically active non-COPD older adults participated in this investigation (Table 1). COPD subjects had been previously diagnosed by a pulmonologist and were clinically stable. Participants were screened if they were aged ≥ 65 years, community dwelling, and reported no participation in a regular resistance training program in the previous 6 months. The subjects completed a medical history questionnaire and performed the short physical performance battery (SPPB) (Guralnik et al., 1994) to assess their physical function. Exclusion criteria included a SPPB score < 4, severe cognitive impairment [mini-mental state examination (MMSE) score < 20], neuromuscular or joint injury, stroke, myocardial infarction, or bone fracture in the previous 6 months, uncontrolled hypertension (>200/110 mmHg), or terminal illness. In addition, non-COPD subjects reported regular participation (≥3 days week^-1^) in exercise activities such as walking, cycling, swimming, or running. All the subjects gave their informed consent and the study was performed in accordance with the Helsinki Declaration and approved by the Ethical Committee of the Toledo Hospital (Spain).

**Table 1:** Comparison of main characteristics of the study participants.

	COPD group			Control group			
	Mean	±	*SD*	Mean	±	*SD*	*p*
Age (years)	78.8	±	7.1	76.6	±	4.1	0.159
BMI (kg ⋅ m^-2^)	30.7	±	6.0	30.1	±	4.0	0.751
SPPB score	10.3	±	2.1	11.8	±	0.4	<**0**.**001**
Habitual gait speed (m ⋅ s^-1^)	1.0	±	0.3	1.2	±	0.4	**0**.**022**
VL muscle thickness (cm)	1.84	±	0.39	1.86	±	0.21	0.877
VL pennation angle (°)	12.7	±	4.5	11.8	±	1.1	0.364
VL fascicle length (cm)	9.9	±	4.1	9.1	±	1.0	0.139

*BMI, body mass index; SPPB, short physical performance battery; VL, vastus lateralis. Bold values indicate significant differences between groups (*p* < 0.05)*.

#### Measurements

Physical function was assessed by means of the 4-m habitual gait speed test. After the cue “ready, set, go!” the subjects walked at their habitual gait speed and the time taken to walk 4 m was recorded. The subjects were asked to walk slightly farther than 4 m in order to avoid deceleration just before covering the 4-m distance.

**Figure 1:**
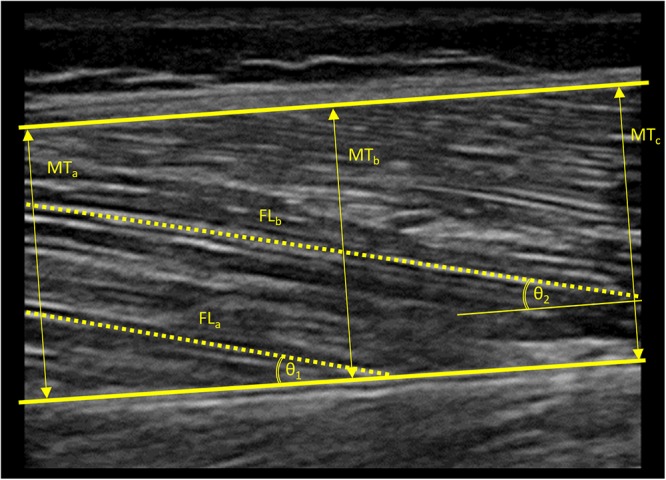
Ultrasound image obtained from the vastus lateralis muscle of a standard subject. Thick lines indicate the superior and inferior aponeuroses of the vastus lateralis muscle. Arrows indicate the three different points at which muscle thickness was measured. Dashed lines indicate the visible portions of muscle fascicles. Arched lines indicate angles formed between the visible muscle fascicles and the inferior aponeurosis. Non-visible portions of muscle fascicles were estimated using a linear extrapolation of fibers and aponeuroses in order to estimate fascicle length. MT, muscle thickness; FL, fascicle length; 𝜃, pennation angle.

Vastus lateralis (VL) muscle thickness and muscle architecture were assessed using B-mode ultrasonography (MyLab 25, Esaote Biomedica, Genova, Italy), with a 50 mm, 10–15 MHz, linear-array probe (Figure 1). Resting ultrasound images were taken at the midpoint of the distance between the superior border of the greater trochanter and the inferior border of the lateral epicondyle of the right leg, with the participant lying on his/her back and the knee slightly flexed at 150° (full knee extension is 180°). The transducer was aligned in the fascicle plane to be able to visualize an optimal portion of fascicles on the ultrasound screen. Then, the images were analyzed by means of image analysis software (ImageJ 1.51q8, NIH, Bethesda, MD, United States). Muscle thickness was measured as the average of the perpendicular distance between the superficial and deep aponeuroses of the VL muscle at three different points on the image (left border, midpoint, and right border). VL pennation angle (PA) and fascicle length (FL) were measured from the visible portion of two fascicles within the same image. In some cases, a small portion of the fascicle extended off the ultrasound window and it was necessary to estimate this non-visible portion using a linear extrapolation of fibers and aponeuroses (Erskine et al., 2009). All the images were collected and digitally analyzed by the same operator. Minimal pressure was applied to the skin during ultrasound assessments and an optimal amount of ultrasound coupling gel was used to ensure image quality. Test–retest coefficients of variation (CV) for muscle architecture measures in our laboratory have previously been reported: 1.9–3.6% (Alegre et al., 2014).

The F–V relationship was assessed in all the subjects after attending two separate familiarization sessions with the leg press equipment (Element+, Technogym, Italy). Then, after a 5-min warm up on a cycle-ergometer (Ergoline, 800 S, Bitz, Germany) and a specific warm up (3 × 10 repetitions at 40% of body mass), the subjects performed two sets of two repetitions with increasing loads (10-kg increments) starting from 40% of their body mass. When the subjects failed to lift a certain load, it was decreased by 5 kg until the 1RM was achieved. Force and velocity data of each repetition were recorded with a linear position transducer (T-Force System, Ergotech, Spain) and a linear regression equation was fitted to the data by means of an Excel^®^ spreadsheet as previously reported (Alcazar et al., 2017). Resting periods between sets were allowed based on the velocity of the preceding repetition (>0.50 m ⋅ s^-1^: 60 s of recovery time; 0.30–0.50 m ⋅ s^-1^: 90 s of recovery time; <0.30 m ⋅ s^-1^: 120 s of recovery time). These rest intervals were adequate to allow the older subjects to perform the second and third repetitions of each set without fatigue, since the highest mean velocity was most frequently exerted during the second repetition (50%), followed by the first repetition (36%) and the third repetition (14%; only performed when necessary, see Alcazar et al., 2017). All subjects were verbally encouraged during testing and all the repetitions were performed as fast and strongly as possible. Mean and instantaneous force and velocity values were obtained in order to conduct the data analysis. Test–retest CV of this F–V testing protocol has been reported to be 4.4–8.5% (Alcazar et al., 2017).

### Data Analysis

Mean force and velocity values from the first repetition of each set were recorded to build the only-concentric F–V relationship, since they were preceded by a resting state. Mean force and velocity values from the second repetition of each set were recorded to build the eccentric–concentric F–V relationship, since they were preceded by an active lengthening of muscle. In both cases, force at zero velocity or the force-intercept (F_0_), velocity at zero force or the velocity-intercept (V_0_), and maximal muscle power (P_max_) were obtained from the F–V regression equations (Alcazar et al., 2017). Using this procedure, we also obtained force and power values exerted at different contraction velocities (as a percentage of concentric V_0_). SSC-induced force/power potentiation at varying contraction velocities was calculated as the relative difference between only-concentric and eccentric–concentric force/power values at the same absolute contraction velocities. In addition, to elucidate the possible mechanisms contributing to SSC-induced power potentiation (a force-related mechanism vs. a velocity-related mechanism), instantaneous force, velocity, and power values across the range of movement during an only-concentric and an eccentric–concentric repetition were analyzed at a low intensity (41.2 ± 10.5% of 1RM) and at a high intensity (81.5 ± 7.2% of 1RM).

### Statistical Analysis

All data were examined for normality of distribution with the Shapiro–Wilk’s test, and log-transformed in case of a non-uniformity result. Standard descriptive statistics were used for continuous variables. The main characteristics and muscle architecture measures were compared between COPD and non-COPD participants by Student’s *t*-tests for independent samples. Significant condition (concentric vs. eccentric–concentric) × group (COPD vs. non-COPD) interactions in SSC-induced potentiation at different intervals of concentric V_0_ were assessed by two-way ANOVA tests. When sphericity was violated, the Greenhouse–Geisser correction was applied. Pairwise comparisons were performed using the Bonferroni test. In addition, within-group differences between instantaneous force, velocity, and power values obtained from a concentric and an eccentric–concentric repetition were assessed with Student’s *t*-tests for dependent samples. Pearson’s correlation was used to assess the association of SSC-induced power potentiation with physical function, muscle thickness, and muscle architecture. Statistical analyses were performed using SPSS v20 (SPSS Inc., Chicago, IL, United States) and the alpha level was set at 0.05.

## Results

No differences in age or body mass index (BMI) were found between COPD and non-COPD older subjects (*p* > 0.05) (Table 1). As expected, non-COPD subjects showed increased SPPB scores and habitual gait speed values compared with the COPD group (both *p* < 0.05). No between-group differences were reported in terms of VL muscle architecture (*p >* 0.05).

**Figure 2:**
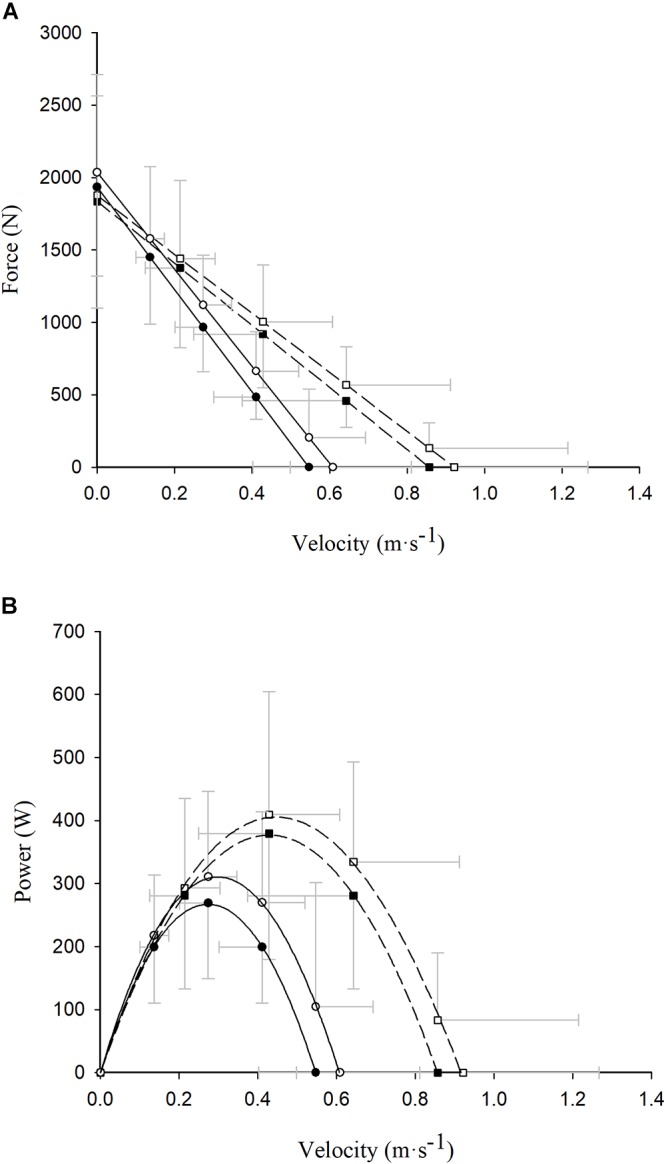
Read figures from up to down. Differences between concentric (closed symbols) and eccentric–concentric (open symbols) force **(A)** and power **(B)** values exerted at different absolute contraction velocities in COPD (circle and continuous line) and healthy older (square and dashed line) participants. Data reported as mean ±*SD*.

The concentric and eccentric–concentric F–V and power–velocity (P–V) relationships of the participants are displayed in Figure 2. No between-group differences were found in terms of concentric F_0_ values (COPD: 1949.4 ± 621.2 N vs. non-COPD: 1834.9 ± 736.0 N; *p* > 0.05), while non-COPD participants showed greater concentric V_0_ (0.86 ± 0.36 m ⋅ s^-1^) and P_max_ (379.2 ± 200.1 W) values compared with the COPD group (0.54 ± 0.15 m ⋅ s^-1^ and 267.7 ± 120.4 W, respectively) (both *p* < 0.05). In contrast, no between-group differences were observed in terms of eccentric–concentric F_0_ (COPD: 2045.8 ± 610.4 N and non-COPD: 1876.4 ± 687.1 N) or P_max_ (COPD: 323.6 ± 143.7 W and non-COPD: 412.9 ± 194.4 W) values (both *p* > 0.05), while significant differences existed for eccentric–concentric V_0_ values (COPD: 0.63 ± 0.21 m ⋅ s^-1^ and non-COPD: 0.90 ± 0.35 m ⋅ s^-1^; *p* < 0.01).

### SSC-Induced Potentiation in the F–V Relationship

Significant condition × group interactions were observed at 50, 60, and 70% of V_0_ for the F–V relationship (all *p* < 0.05) (Table 2). Pairwise comparisons showed that the SSC increased force values compared with only-concentric repetitions at all 10% intervals of V_0_ (i.e., 0–100% of V_0_) among COPD subjects (all *p* < 0.05), while the same was observed among non-COPD older participants only between 40 and 90% of V_0_ (all *p* < 0.05). No significant differences were reported between COPD and non-COPD subjects regarding force production at the different V_0_ intervals for any test condition (i.e., concentric and eccentric–concentric) (*p* > 0.05).

**Table 2:** Comparison of concentric and eccentric–concentric force values at various contraction velocities relative to maximal concentric unloaded shortening velocity.

	COPD group						Control group						ANOVA interaction
	CON			SSC			CON			SSC			
V_0_ (%)	Mean	±	*SD*	Mean	±	*SD*	Mean	±	*SD*	Mean	±	SD	
0	1949.4	±	621.2	2045.8	±	610.4^∗^	1834.9	±	736.0	1876.4	±	687.1	0.483
1	1929.9	±	614.9	2027.8	±	604.6^∗^	1816.5	±	728.6	1859.0	±	681.1	0.472
10	1754.4	±	559.0	1865.4	±	552.1^∗^	1651.4	±	662.4	1701.9	±	627.3	0.363
20	1559.5	±	496.9	1685.1	±	494.9^∗^	1467.9	±	588.8	1527.3	±	567.9	0.235
30	1364.6	±	434.8	1504.7	±	439.1^∗^	1284.4	±	515.2	1352.7	±	509.2	0.123
40	1169.6	±	372.7	1324.4	±	385.6^∗^	1100.9	±	441.6	1178.1	±	451.3^∗^	0.055
50	974.7	±	310.6	1144.1	±	335.3^∗^	917.4	±	368.0	1003.5	±	394.5^∗^	**0.030**
60	779.8	±	248.5	963.7	±	289.9^∗^	734.0	±	294.4	828.9	±	339.5^∗^	**0.030**
70	584.8	±	186.3	783.4	±	252.0^∗^	550.5	±	220.8	654.3	±	287.4^∗^	**0.048**
80	389.9	±	124.2	603.0	±	225.6^∗^	367.0	±	147.2	479.8	±	239.8^∗^	0.080
90	194.9	±	62.1	422.7	±	214.8^∗^	183.5	±	73.6	305.2	±	200.2^∗^	0.120
99	19.5	±	6.2	260.4	±	218.6^∗^	18.3	±	7.4	148.0	±	175.9	0.159
100	0	±	0	242	±	221^∗^	0.0	±	0.0	130.6	±	174.1	0.163

*CON, concentric; SSC, eccentric–concentric; V_*0*_, maximal unloaded shortening velocity; SD, standard deviation.^∗^Significant differences compared with CON values (*p* < 0.05). Bold values indicate a significant ANOVA interaction (*p* < 0.05)*.

### SSC-Induced Potentiation in the P–V Relationship

No significant condition × group interactions were reported at the different V_0_ intervals for the P–V relationship (*p* > 0.05) (Table 3). However, the SSC led to increased power values compared with only-concentric repetitions at all 10% intervals of V_0_ (except at 0% of V_0_) among COPD participants, and between 30 and 90% of V_0_ among non-COPD participants (all *p* < 0.05). On the other hand, significant differences between COPD and non-COPD older subjects were noted in the concentric P–V relationship between 1 and 99% of V_0_ (all *p* < 0.05), while between-group differences in the eccentric–concentric P–V relationship were only noted at 1% of V_0_ (*p* < 0.05).

**Table 3:** Comparison of concentric and eccentric–concentric power values at various contraction velocities relative to maximal concentric unloaded shortening velocity.

	COPD group						Control group						ANOVA interaction
	CON			SSC			CON			SSC			
V_0_ (%)	Mean	±	*SD*	Mean	±	*SD*	Mean	±	*SD*	Mean	±	SD	
0	0.0	±	0.0	0.0	±	0.0	0.0	±	0.0	0.0	±	0.0	–
1	10.6	±	4.8	11.1	±	4.7^∗^	15.0	±	7.9^¥^	15.4	±	7.6^¥^	0.788
10	96.4	±	43.3	102.1	±	43.5^∗^	136.5	±	72.0^¥^	140.6	±	69.1	0.688
20	171.3	±	77.1	184.4	±	78.2^∗^	242.7	±	128.0^¥^	251.9	±	123.0	0.545
30	224.9	±	101.1	246.9	±	104.4^∗^	318.6	±	168.0^¥^	333.7	±	161.9^∗^	0.376
40	257.0	±	115.6	289.8	±	122.5^∗^	364.1	±	192.1^¥^	386.1	±	186.0^∗^	0.228
50	267.7	±	120.4	312.8	±	133.1^∗^	379.2	±	200.1^¥^	409.1	±	195.3^∗^	0.159
60	257.0	±	115.6	316.2	±	137.3^∗^	364.1	±	192.1^¥^	402.7	±	190.6^∗^	0.164
70	224.9	±	101.1	299.8	±	136.8^∗^	318.6	±	168.0^¥^	367.0	±	172.7^∗^	0.206
80	171.3	±	77.1	263.6	±	134.8^∗^	242.7	±	128.0^¥^	301.8	±	144.6^∗^	0.258
90	96.4	±	43.3	207.7	±	136.4^∗^	136.5	±	72.0^¥^	207.2	±	114.5^∗^	0.307
99	10.6	±	4.8	140.5	±	146.3^∗^	15.0	±	7.9^¥^	96.9	±	105.7	0.345
100	0.0	±	0.0	132.1	±	148.1^∗^	0.0	±	0.0	83.2	±	107.0	0.349

*CON, concentric; SSC, eccentric–concentric; V_*0*_, maximal unloaded shortening velocity; SD, standard deviation.^∗^Significant differences compared with CON values (*p* < 0.05). ^¥^Significant differences compared with the COPD group (*p* < 0.05)*.

### Force-Related vs. Velocity-Related SSC-Induced Potentiation in Muscle Power

**Figure 3:**
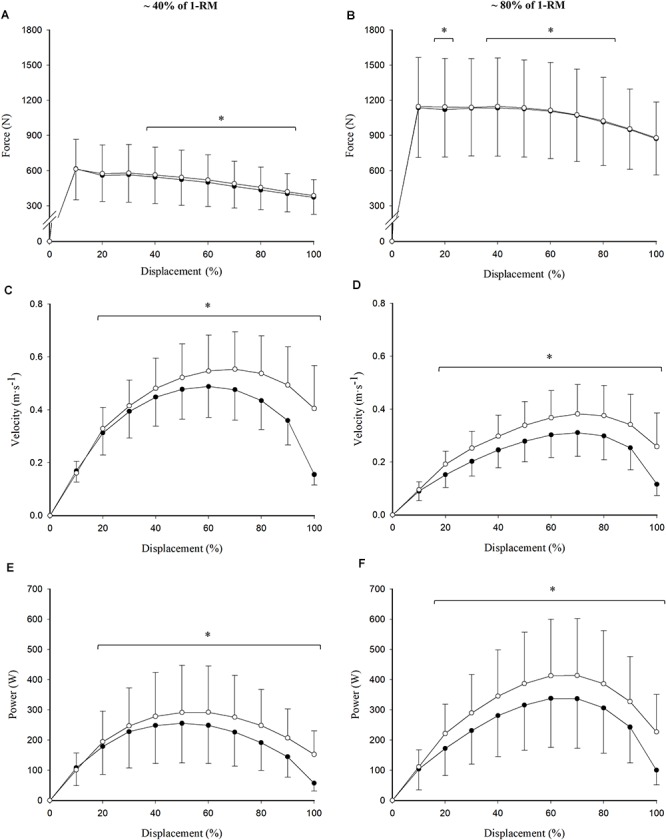
Read figures from up to down and from left to right. Differences between concentric (closed circles) and eccentric–concentric (open circles) force **(A,B)**, velocity **(C,D)**, and power **(E,F)** values across the range of movement at a low (41.2 ± 10.5% of 1RM; **A,C,E**) and a high (81.5 ± 7.2% of 1RM; **B,D,F**) intensity. Data reported as mean ± standard deviation. ^∗^Significant differences between concentric and eccentric–concentric values (*p* < 0.05).

Loads equivalent to 41.2 ± 10.5% (0.675 ± 0.145 s of CON duration) and 81.5 ± 7.2% (0.914 ± 0.249 s of CON duration) of 1RM were selected as representative of the high-force/low-velocity and low-force/high-velocity regions of the F–V relationship, respectively. Differences were observed in terms of force production at different points on the range of movement between the concentric and the eccentric–concentric repetitions (Figure 3A,B). While these differences seemed to be located in the mid-portion (40–90%) of the range of movement with the low load (i.e., ∼40% of 1RM), they were distributed slightly more toward the beginning of the movement (20 and 40–80%) in the high-load condition (i.e., ∼80% of 1RM) (all *p* < 0.05). Velocity (Figure 3C,D) and power (Figure 3E,F) differences between the concentric and the eccentric–concentric repetitions also reached statistical significance for both the low- and the high-load conditions between 20 and 100% of range of movement (all *p* < 0.05). These differences were similarly distributed along the range of movement at both intensities, except at the beginning of the movement (0–10%), where significant differences were not observed in any case (*p* > 0.05).

### Association Between SSC-Induced Potentiation and Other Functionally Relevant Variables

Habitual gait speed showed a negative association with SSC-induced power potentiation among COPD participants. Specifically, the associations reached statistical significance at 50, 60, 70, 80, and 90% of V_0_ (*r* = -0.40 to -0.50; all *p* < 0.05). A negative association was also observed between VL muscle thickness and SSC-induced power potentiation at 80, 90, and 99% of V_0_ (*r* = -0.44 to -0.49; all *p* < 0.05). Furthermore VL PA was found to be negatively associated with SSC-induced power potentiation at 70, 80, 90, and 99% of V_0_ (*r* = -0.43 to -0.54; all *p* < 0.05). Finally, no significant correlations were found between VL FL and SSC-induced power potentiation.

## Discussion

Our main findings were: (1) older people with COPD showed lower levels of concentric P_max_ compared with age-matched physically active older adults without COPD; (2) COPD participants exhibited a greater SSC-induced potentiation in force values compared with non-COPD subjects; (3) between-group differences in P_max_ disappeared after the inclusion of a SSC muscle action; and (4) the SSC-induced potentiation was negatively associated with physical function, muscle thickness, and PA among COPD older subjects.

Limb muscle dysfunction is a systemic consequence of COPD characterized by the presence of a decreased proportion of type I muscle fibers (Gosker et al., 2007) and oxidative capacity (Green et al., 2008a), and reduced muscle cross-sectional area, maximal strength, and endurance (Couillard et al., 2002, 2003). In addition, we observed a 22% lower concentric P_max_ in COPD compared with non-COPD older subjects, which was fundamentally caused by a deficit in the velocity component of muscle power or the ability to produce force at relatively high contraction velocities. This fact contrasts with a particular characteristic of limb muscle dysfunction in COPD, in which in contrast with normal aging, people with COPD experience a shift in fiber type distribution from type I fibers to type II fibers (Gosker et al., 2007). However, single muscle fibers of COPD patients may present altered excitation–contraction coupling (Green et al., 2008b) and decreased ATPase activity compared with age-matched healthy controls (Stubbings et al., 2008), contributing to the slowing-down of the F–V relationship observed in the present investigation.

**Figure 4:**
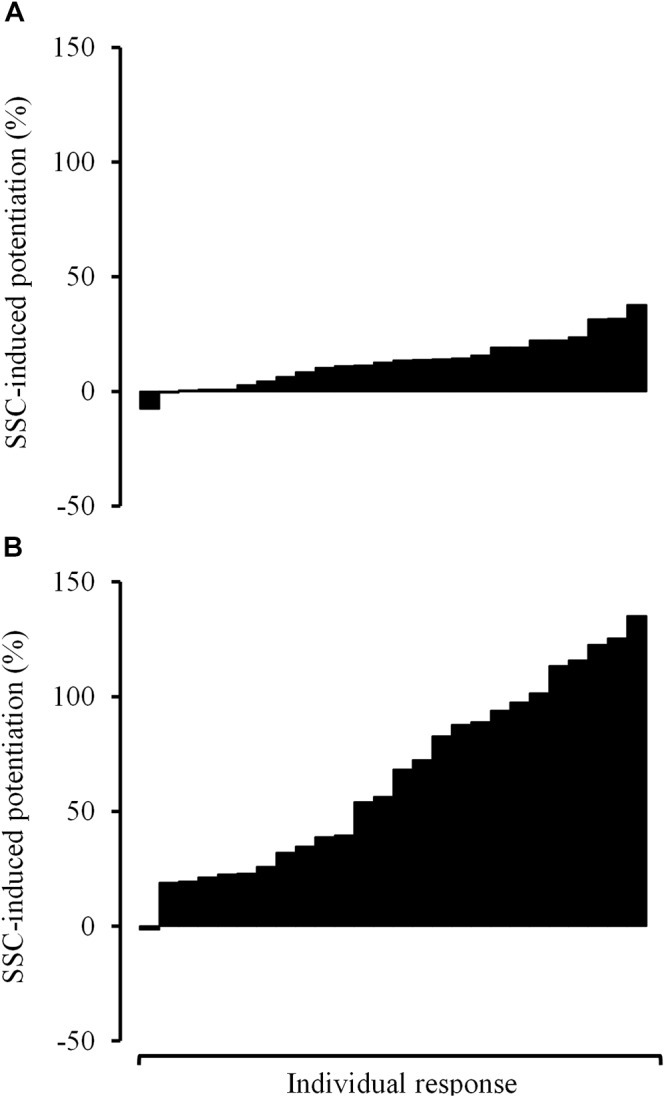
Read figures from up to down. Individual response observed among COPD participants in terms of stretch-shortening cycle-induced potentiation in force values at 20% of V_0_ **(A)** and 80% of V_0_ **(B)**. V_0_, maximal unloaded shortening velocity.

On the other hand, the inclusion of the SSC within repetitions led to greater improvements in force in COPD compared with non-COPD older subjects, especially among those contraction velocities eliciting the highest power values in the eccentric–concentric F–V relationship (i.e., 50–70% of concentric V_0_). This fact partially compensated previous differences in concentric P_max_, so that differences in eccentric–concentric P_max_ did not reach the statistical significance. Nevertheless, a relatively moderate variability in the rate of SSC-induced potentiation was observed among subjects (Figure 4). A higher variability in the effect induced by the SSC in older adults in comparison with younger subjects has previously been reported (Bosco and Komi, 1980). This variability may be explained by the different mechanisms involved in the SSC-induced potentiation that have been suggested in the literature: increased neural drive, tendon recoil, and contractile potentiation (Cohen, 1988; Hopkins et al., 2009).

Although increased muscle excitation levels have been observed during the 200 ms before the start of the concentric phase of a SSC action compared with a purely concentric one (Walshe et al., 1998), there is evidence showing a similar neural drive to contracting muscles during the concentric phase of a purely concentric and a SSC muscle action (Walshe et al., 1998; Finni et al., 2003). However, other different mechanisms might explain the SSC-induced force increment observed during a concentric muscle action. Tendons are visco-elastic structures that exhibit both rate-dependent (viscous) and rate-independent (elastic) properties. During a SSC action, tendons undergo initial lengthening in the early eccentric phase, with smaller changes in length in the late eccentric phase (Earp et al., 2017). Additional lengthening is experienced by tendons in the early concentric phase until skeletal muscle force peaks and tendons reach their peak length (Earp et al., 2017). After that, tendons start shortening and release elastic energy (Finni et al., 2003; Earp et al., 2017), that can be as high as 85% of that stored during previous lengthening (Shadwick, 1990). Thus, the contribution of tendon shortening to muscle-tendon complex velocity has been reported to be 50% in some cases, although this proportion varied among subjects (Chino et al., 2008). Because of this mechanism of tendon recoil, the contraction velocity of muscle fascicles is reduced for a given muscle-tendon unit velocity (Finni et al., 2003). Therefore, muscle fascicles can produce a greater amount of force at a corresponding joint velocity according to the F–V relationship of skeletal muscles. This mechanism might be enhanced by other structures within muscles that may exhibit spring-like properties (such as actomyosin cross-bridges, actin and myosin filaments, titin, and the connective tissue scaffolding of the extracellular matrix) (Roberts, 2016). In addition, residual force enhancement after stretching has been associated with active and passive muscle contraction-related mechanisms (Rassier and Herzog, 2004). It has been demonstrated that thick filaments are switched on by mechanical stress (Irving, 2017). The increase in the proportion of attached cross-bridges caused by the mechanical stress imposed over the thick filaments (Irving, 2017) and the contributing role of the protein titin to active force production (Herzog et al., 2016) might both explain the increased force production during the early phase of a concentric muscle action preceded by an eccentric muscle action. Furthermore, SSC-induced power potentiation seems to be highly dependent on the fast transition from the eccentric to the concentric muscle action (Newton et al., 1997; Schmidtbleicher, 1997; Lappin et al., 2006). Perhaps this is the reason for the differences observed in the magnitude of the SSC-induced power potentiation values across the F–V relationship, with the effect of the SSC being relatively greater for the fastest muscle actions (i.e., lower contraction times).

Surprisingly, a higher SSC-induced power potentiation was associated with lower physical function, VL muscle thickness, and VL PAs in our sample of older people with COPD. Moreover, physically active non-COPD older subjects showed higher physical function values and lower SSC-induced potentiation than COPD subjects. In order to confirm these results, we looked at the data obtained in another study previously published by our research group with community-dwelling older adults (Alcazar et al., 2017), and observed a statistically significant negative association between SSC-induced power potentiation at 60% of 1RM and habitual gait speed (*r* = -0.43; *p* < 0.05; *n* = 31; unpublished results). Unfortunately, the eccentric–concentric F–V relationship was not assessed in that study, and so we could not include those subjects in the present investigation. Therefore, the SSC-induced power potentiation seems to be a negative outcome in older people. Impaired muscle excitation of the agonist muscles has been reported to be a major cause of mobility limitations in older people (Clark et al., 2010). The longitudinal decrement in muscle excitation levels among older subjects has been observed to be as high as 9% per year, and has been strongly associated with the loss of muscle power with aging (Clark et al., 2013). A possible explanation for the negative association between SSC-induced power potentiation and physical function is that they both share the same characteristic: decreased concentric muscle function. Muscle excitation levels during a solely concentric muscle action are severely impaired in older subjects with mobility limitations, which may also be associated with decreased muscle thickness and PA values. This concentric deficit might be compensated during a SSC muscle action by the effects of tendon recoil and the potentiation of the contractile material within skeletal muscles, and thus the mobility limited older subject would exhibit a greater power generating capacity during the SSC muscle actions compared with the purely concentric muscle actions. Actually, there is strong evidence supporting the specific and strong age-related deficit in maximal concentric force, which is progressively higher at faster contraction velocities (Thom et al., 2007), while maximal eccentric force values seem to be better preserved with age (Roig et al., 2010), especially in COPD individuals (Mathur et al., 2007). In fact, the relatively preserved eccentric force values observed in older people have been associated with enhanced residual force enhancement after active stretching (Power et al., 2012). Thus, the augmented SSC-induced response might be a compensatory mechanism to produce muscle power in those older subjects with an impaired physical function and reduced muscle size and PA values. This SSC-related compensatory mechanism has also been reported to be involved in the attenuated loss of muscle function observed during countermovement compared with squat jumps after exercise-induced muscle damage in young subjects (Byrne and Eston, 2002).Unfortunately, the tendon properties and muscle excitation levels of the participants were not assessed in the present investigation in order to find the possible mechanisms leading to the enhanced SSC-induced response observed among COPD individuals. However, we should highlight that this is the first study in which the effect of the SSC on the F–V relationship of older people (with and without COPD) has been investigated. Future studies are warranted in order to elucidate the actual mechanisms involved in the SSC-induced power potentiation observed in mobility-limited older subjects with COPD.

## Conclusion

Older adults with COPD produced lower concentric P_max_ values than non-COPD subjects as a consequence of a diminished capacity to produce force at relatively high contraction velocities. The SSC was found to enhance muscle power production, especially among older subjects with COPD, eliminating differences in eccentric–concentric P_max_ values between COPD and non-COPD participants. Finally, SSC-induced potentiation was negatively associated with physical function, VL muscle thickness, and PA, but not with VL FL. Future studies should be conducted in order to determine the mechanisms that explain a greater SSC-induced potentiation in mobility-limited older adults with COPD.

## Data Availability

The datasets for this study will not be made publicly available because we are unable to provide the minimal dataset because these data are a part from a big project that are pending to be published. While the main paper is waiting, you can request any doubt from the data to the corresponding author via e-mail: Luis.Alegre@uclm.es.

## Author Contributions

RN-C, JA, CR-L, JL-R, AA-A, IA, FG-G, and LA conceived and designed the experiments and interpreted the results of research. RN-C, JA, CR-L, JL-R, and FG-G performed the experiments. RN-C, JA, IA, LA, A-AA, and CR-L analyzed the data.RN and JA drafted manuscript and prepared tables/figures. RN-C, JA, CR-L, JL-R, AA-A, IA, FG-G, and LA edited and critically revised the paper and approved the final version of the manuscript.

## Conflict of Interest Statement

The authors declare that the research was conducted in the absence of any commercial or financial relationships that could be construed as a potential conflict of interest.
